# Nationwide cost-effectiveness and quality of life analysis of minimally invasive distal pancreatectomy

**DOI:** 10.1007/s00464-024-10849-0

**Published:** 2024-08-20

**Authors:** Matteo De Pastena, Alessandro Esposito, Salvatore Paiella, Greta Montagnini, Caterina C. Zingaretti, Marco Ramera, Danila Azzolina, Dario Gregori, Emanuele F. Kauffmann, Alessandro Giardino, Luca Moraldi, Giovanni Butturini, Ugo Boggi, Roberto Salvia

**Affiliations:** 1 General and Pancreatic Surgery Department, Pancreas Institute, University and Hospital Trust of Verona, Verona, Italy; 2 Department of Clinical and Experimental Sciences, University of Brescia, Brescia, Italy; 3 Department of Environmental and Preventive Science, University of Ferrara, Ferrara, Italy; 4 Unit of Biostatistics, Epidemiology, and Public Health, Department of Cardiac, Thoracic, and Vascular Sciences, University of Padova, Padova, Italy; 5 Division of General and Transplant Surgery, Pisa University Hospital, Pisa, Italy; 6 HPB Surgery Unit, Pederzoli Hospital, Peschiera del Garda, Verona, Italy; 7 Division of Oncologic Surgery and Robotics, Department of Oncology, Careggi University Hospital, Florence, Italy; 8 University of Verona, Verona, Italy; 9 Unit of General and Pancreatic Surgery – The Pancreas Institute Verona, Department of Engineering for Innovation Medecine, University of Verona, Verona, Italy

**Keywords:** Robotic distal pancreatectomy, Laparoscopic distal pancreatectomy, Cost analysis, Minimally invasive pancreatectomy, Pancreatic surgery

## Abstract

**Background:**

This study analyzed the Quality of Life (QoL) and cost-effectiveness of laparoscopic (LDP) versus robotic distal pancreatectomy (RDP).

**Methods:**

Consecutive patients submitted to LDP or RDP from 2010 to 2020 in four high-volume Italian centers were included, with a minimum of 12 months of postoperative follow-up were included. QoL was evaluated using the EORTC QLQ-C30 and EQ-5D questionnaires, self-reported by patients. After a propensity score matching, which included BMI, gender, operation time, multiorgan and vascular resections, splenic preservation, and pancreatic stump management, the mean differential cost and Quality-Adjusted Life Years (QALY) were calculated and plotted on a cost-utility plane.

**Results:**

The study population consisted of 564 patients. Among these, 271 (49%) patients were submitted to LDP, while 293 (51%) patients to RDP. After propensity score matching, the study population was composed of 159 patients in each group, with a median follow-up of 59 months. As regards the QoL analysis, global health and emotional functioning domains showed better results in the RDP group (*p* = 0.037 and *p* = 0.026, respectively), whereas the other did not differ. As expected, the median crude costs analysis confirmed that RDP was more expensive than LDP (16,041 Euros vs. 10,335 Euros, *p* < 0.001). However, the robotic approach had a higher probability of being more cost-effective than the laparoscopic procedure when a willingness to pay more than 5697 Euros/QALY was accepted.

**Conclusion:**

RDP was associated with better QoL as explored by specific domains. Crude costs were higher for RDP, and the cost-effectiveness threshold was set at 5697 euros/QALY.

In the current trend of economic recession, limited resources, and medical expense control, the cost-effectiveness of a novel procedure or the surgical technique cannot be underestimated at the time of its introduction. The diffusion of the minimally invasive approach, even for pancreatic surgery, has progressively increased, especially for the resection of the distal pancreas [1]. This led to several cost-analysis to assess the benefit and sustainability of the new technique over the standard open procedure. Actually, the minimally invasive approach to the distal pancreatectomy (MIDP) is considered the gold-standard for the treatment of left pancreatic lesions [2].

MIDP has been widely reported in the literature to result in lower blood loss, higher rates of splenic preservation, decreased postoperative morbidity and decreased length of stay when compared to open distal pancreatectomy (ODP) [3, 4]. MIDP has proven to be effective for pancreatic cancers as well.

However, the choice between the two minimally invasive approaches, laparoscopic or robotic, has remained a subject of ongoing debate. While both techniques offer potential benefits in terms of clinical outcomes, it is crucial to assess not only their efficacy but also their cost-effectiveness and impact on patients’ quality of life. Understanding the economic and patient-centric aspects of these surgical approaches is vital, as it can guide healthcare providers, patients, and policymakers in making informed decisions regarding the most suitable method for distal pancreatectomy.

The study aims to analyze the cost-effectiveness and the Quality of Life (QoL) of the laparoscopic (LDP) versus robotic distal pancreatectomy (RDP) in Italian high-volume pancreatic centers.

## Methods

### Patient population and study design

The study was performed according to the Strengthening the Reporting of Observational Studies in Epidemiology (STROBE) guidelines, while the cost-effectiveness analysis was carried out according to the EVEREST guidelines [5, 6]. The local Institutional Review Board approved the data collection and analysis procedures. The study population consisted of patients who underwent LDP or RDP between 2010 and 2020, for any condition, with at least 12 months of follow-up, at four high-volume Italian centers of minimally invasive pancreatic surgery. All data were retrieved from the institutional electronic and prospectively maintained database and retrospectively analyzed. The participating centers were Pancreatic Surgery Unit of the Verona Pancreas Institute, Verona, the General Surgery Unit of the Careggi Hospital of Florence, the division of General and Transplant Surgery of the Pisa University Hospital, and the Pancreatic Surgery Unit of Pederzoli Hospital of Peschiera del Garda. All the participating centers are high-volume pancreatic centers, performing both LDP and RDP during the study period.

All cases were reviewed and discussed at a dedicated institutional surgical meeting, where the decision to perform a minimally invasive procedure was discussed among surgeons. Each patient underwent a preoperative contrast-enhanced CT-scan of the abdomen. The decision to pursue a minimally invasive approach was left to the discretion of the individual centers. However, common indications for MIS included benign or pre-malignant lesions smaller than 10 cm, or malignancies without evidence of major involvement of the peripancreatic vessels.

All the centers did not have a dedicated robotic platform for HPB unit. Consequently, the robotic cases were scheduled based on the availability of the Da Vinci Surgical System®.

### Study endpoints

LDP and RDP were compared according to the primary and secondary endpoints set. Primarily, the two techniques were compared for patient QoL at 12 months and cost-efficacy; secondarily, they were compared for the efficacy, using well-known surgical and postoperative metrics (intraoperative blood loss and operative time, and conversion rate), and postoperative outcomes (major complications, length of stay and mortality).

### Data collection and definitions

Baseline characteristics included sex, age, body mass index (BMI, kg/m2), American Society of Anesthesiologists (ASA) physical status, diabetes mellitus, and history of other malignancies.

The surgical data collected were: conversion rate; splenectomy rate; operative time (min); blood loss (mL); any additional organ resection (beyond splenectomy); transection level of the pancreas (divided into the pancreatic neck, gastroduodenal artery level, left border of the aorta or more distal; pancreatic stump management (handsewn, reinforced stapler or ultrasonic scalpel). The following 90-day postoperative complications were considered: major complications (classified as Clavien-Dindo ≥ III) [7]; postoperative pancreatic fistula (POPF) was defined and classified according to ISGPF [8]; delayed gastric emptying (DGE), post-pancreatectomy hemorrhage (PPH), and chyle leak classified according to the ISGPS definition [9–11]; any pulmonary complication; intensive care unit admission; reoperation rate; length of stay (days); 90-day readmission, and 90-day mortality. Pathological data were also recorded, about final histology, tumor size (mm), and the number of lymph nodes harvested

### Surgical procedures

LDP and RDP were performed as already described [12–16]. The Da Vinci Surgical System® (Intuitive Surgical Inc, Sunnyvale CA) was used for all robotic procedures. Splenic preservation was performed selectively and solely in instances of presumed benign or pre-malignant lesions. The site of pancreatic transection was tailored on a case-by-case basis, with the objective of conserving pancreatic tissue in benign lesions rather than undergoing a standard distal pancreatectomy. The pancreatic transection and the management of the pancreatic stump were executed in accordance with the Institution’s policy. The decision of whether to adopt one technique over another was made at the surgeon’s discretion, mainly based on pancreatic thickness. Three distinct techniques were described: stapler, reinforced or not; ultrasonic dissector, operated at the lowest vibration level throughout the pancreatic dissection; and handsewn management of the pancreatic stump following resection of the pancreas with the energy devices. At least one surgical drain was positioned adjacent to the pancreatic remnant; when two drains were placed, the other was placed in the splenic cavity. The drain was managed in the postoperative course in accordance with the previously described early drain removal protocols [17, 18].

### QoL analysis

All patients included in the study had at least 12 months of follow-up. Data regarding patients’ QoL were obtained from two different self-administered questionnaires: a condition-specific questionnaire (European Organization for Research and Treatment of Cancer Quality of Life Questionnaire C-30, EORTC QLQ-C30) and a generic questionnaire (Euro QOL five dimensions, EQ-5D). The Italian translated version of the questionnaires was used. The EORTC QLQ-C30 was divided into:

- One global domain:
  o Global health (GH): a measure of overall well-being.
- Five functional scales:
  o Physical functioning (PF): ability to perform activities of daily living.
  o Role functioning (RF): ability to perform social and occupational roles.
  o Emotional functioning (EF): ability to manage emotions and maintain a positive outlook.
  o Cognitive functioning (CF): ability to remember, concentrate, and make decisions.
  o Social functioning (SF): ability to maintain relationships and social activities.
- Eight symptom scales:
  o Fatigue (FA): feeling tired or lacking energy.
  o Pain (PA): experiencing discomfort or pain.
  o Nausea and vomiting (NV): experiencing nausea or vomiting.
  o Dyspnea (DY): difficulty breathing.
  o Appetite loss (AP): having a decreased appetite.
  o Sleep disturbance (SL): having trouble sleeping or feeling unrested.
  o Constipation (CO): having difficulty passing stools.
  o Diarrhea (DI): having loose or watery stools.
- One item exploring the financial impact (FI): a measure of the financial burden of the disease.

The analysis of the questionnaire scores was performed according to the EORTC QLQ-C30 Scoring Manual. The QoL questionnaires (EORTC QLQ-C30 and EQ-5D) were collected after at least 12 months of follow-up from the surgery.

### Cost-effectiveness analysis

The evaluation of the impact of the surgical approach on the quality and quantity of life of the patient underwent MIDP was analyzed by the assessment of the quality-adjusted life years (QALYs). QALY was a composite indicator that merge the quantity and quality of life related to a single index combined to the “utility” parameter. Utilities of health states are generally expressed on a numerical scale ranging from 0 to 1, in which 0 represents the utility of the state “Dead” and 1 the utility of a state lived in “perfect health”. All these variables were extracted from the values of generic preference-based measures (EQ-5D). In order to determine the exact QALY value, it is sufficient to multiply the utility value associated with a given state of health by the years lived in that state [19]. The total costs of the two surgical procedures (LDP and RDP) were divided into intraoperative and postoperative costs and calculated using the hospital expenditure report, then compared. Costs were analyzed in Euros.

Intraoperative costs included the following:

- Operative theater use and maintenance costs (the staff received a fixed salary);
- Anesthesiology costs;
- Surgical instrumentation (including Da Vinci® system costs for the RDP).

The initial purchase expenses of the robotic and laparoscopic systems were excluded.

Postoperative costs were calculated considering the hospitalization costs and including the costs of any postoperative imaging study, any nutritional support, any surgical or interventional postoperative procedure, and the intensive care unit admission expenses. Overall costs were calculated by adding intraoperative and postoperative cost for each patient. The mean differential cost and mean differential QALY were calculated and plotted on a cost-utility plane. The cost-effectiveness was also explored using the incremental cost-effectiveness ratio (ICER), incremental net monetary benefit (INMB), and the cost-effectiveness acceptability curve (CEAC). The Italian gross domestic product (GDP) per capita was considered as a reference value assuming an ICER of 1xGDP to define an intervention as cost-effective. The INMB was calculated to obtain a confidence interval for producing the cost-effectiveness analysis acceptability curve. Otherwise, the CEAC analyzes the probability that an intervention was cost-effective when compared with the standard treatment. The plotted data described the willingness to pay (WTP), which was the range of monetary values that a decision-maker might be willing to pay to obtain a particular unit change (QALY) in the outcome.

### Statistical analysis

To minimize bias, RDP and LDP were compared using a 1:1 propensity score matched strategy Matching was based on specific perioperative variables to equalize the complexity of the surgical cases, including BMI, gender, operation time, multiorgan and vascular resections, splenic preservation rate, and pancreatic stump management. Nearest neighbor matching was performed with a caliper width of 0.01. Data were analyzed on an intention-to-treat basis, including patients who required conversion to a laparotomic procedure. Continuous variables were reported as the median and interquartile range (IQR). Comparative analysis between groups was conducted using χ^2^ or Fisher’s exact tests for categorical variables, while the Mann–Whitney U test was used for continuous variables. A *p*-value < 0.05 was considered statistically significant (two-tailed). Data were analyzed using Statistical Package for the Social Sciences (SPSS) version 24.0 for Windows (SPSS, Inc., Chicago, IL). Statistical software R version 4.2.0 was used for cost-effectiveness analysis.

## Results

### General characteristics

During the study period, 564 patients fulfilled the inclusion and exclusion criteria for minimally invasive DP at the four high-volume centers. Among these, 271 (49%) patients were submitted to LDP, while 293 (51%) patients underwent RDP. At the univariate analysis, significant differences were found between the groups in terms of median age (61 in LDP vs. 57 in RDP, *p* = 0.036) and ASA ≥ 3 (25% in LDP vs. 16% in RDP, *p* = 0.001) (Table 1).

**Table 1:** Demographic and intraoperative data

	Laparoscopic DP	Robotic DP	*p*-value
	(*n* = 271, 48%)	(*n* = 293, 52%)	
Age year, median (IQR)	61 [48–70]	57 [45–68]	**0.036**
Female, *n*° (%)	175 (65%)	186 (64%)	0.428
BMI, kg/m^2^, median (IQR)	24 [22–27]	24 [22–27]	0.615
ASA ≥ 3, *n*° (%)	68 (25%)	48 (16%)	**0.001**
Comorbidities, *n*° (%)	183 (68%)	204 (70%)	0.328
Previous Abdominal surgery, *n*° (%)	115 (42%)	106 (36%)	0.076
Neoadjuvant therapy, *n*° (%)	14 (5%)	8 (3%)	0.101
Conversion rate	38 (14%)	12 (4%)	** < 0.001**
Spleen preserving	56 (21%)	93 (32%)	**0.002**
Multiorgan resection, *n*° (%)	43 (16%)	28 (10%)	**0.016**
Vascular resection, *n*° (%)	10 (4%)	11 (4%)	0.573
Intraoperative transfusion, *n*° (%)	15 (6%)	9 (3%)	0.108
Pancreatic stump management			**0.002**
Stapler	205 (75%)	188 (64%)	
Ultrasonic Scalpel	56 (21%)	76 (26%)	
Hand-sewn	10 (4%)	29 (10%)	
EBL ml, median (IQR)	100 [100–200]	100 [100–150]	0.289
OT min, median (IQR)	250 [200–320]	285 [230–365]	** < 0.001**

Bold value indicate statistical significance (*p* < 0.05)

*BMI* body mass index, *ASA* American Society of Anesthesiology, *OT* operation time, *EBL* estimated blood loss

### Procedure-related expenditures comparison and cost-effectiveness analysis

The crude costs of the two procedures were calculated by dividing the total costs into intraoperative and postoperative costs (Table 2). As expected, the comparison of the intraoperative expenditures confirmed that the robotic approach was more expensive than the laparoscopic one (7030 ± 5106 vs. 4178 ± 6309 Euros, *p* < 0.001). On the other hand, the postoperative costs were not significantly different (6666 ± 6963 vs. 7512 ± 5888, *p* = 0.021). However, when analyzing the total cost of the hospitalization, it was again verified that the expenses of RDP were higher than LDP (16,967 ± 8508 vs. 10,844 ± 12,547 Euros, *p* < 0.001).

**Table 2:** Cost-effectiveness analysis

	Before propensity score-matching			After propensity score-matching		
	Laparoscopic DP	Robotic DP	*p*-value	Laparoscopic DP	Robotic DP	*p*-value
	(*n* = 271, 48%)	(*n* = 293, 52%)		(*n* = 159)	(*n* = 159)	
QALY median (IQR)	0.73 [0.61–1.0]	1.0 [0.69–1.0]	**0.026**	0.75 [0.64–1.0]	1.0 [0.71–1.0]	**0.031**
Intra-operative Costs, Euros, (± SD)	4178 [± 6309]	7030 [± 5106]	** < 0.001**	2939 [± 1697]	8870 [± 7545]	**0.002**
Post-operative Costs, Euros, (± SD)	6666 [± 6963]	7512 [± 5888]	0.119	6027 [± 7295]	7749 [± 6763]	0.200
Total Costs, Euros, (± SD)	10,844 [± 12,547]	16,967 [± 8508]	** < 0.001**	10,335 [± 7202]	16,041 [± 6608]	** < 0.001**

Bold value indicate statistical significance (*p* < 0.05)

**Fig. 1:**
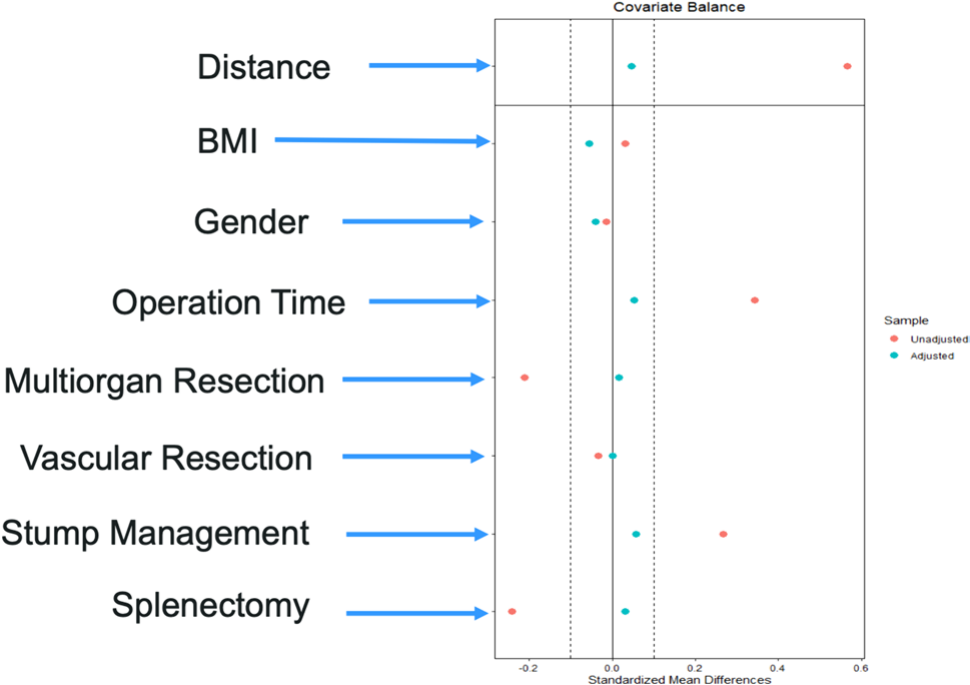
The propensity score matching plot. The study population consisted of 418 patients, 209 each group

A propensity score matching was performed to balance the cohort. The parameters included were BMI, gender, operation time, multiorgan and vascular resections, spleen-preserving rate, and management of the pancreatic stump, as shown in Fig. 1. After matching the cohort, the study population consisted of 318 patients. The postoperative expenses were still balanced (*p* = 0.200). However, the intraoperative and total costs were still higher in the RDP group (*p* = 0.002 and *p* < 0.001, respectively).

**Fig. 2:**
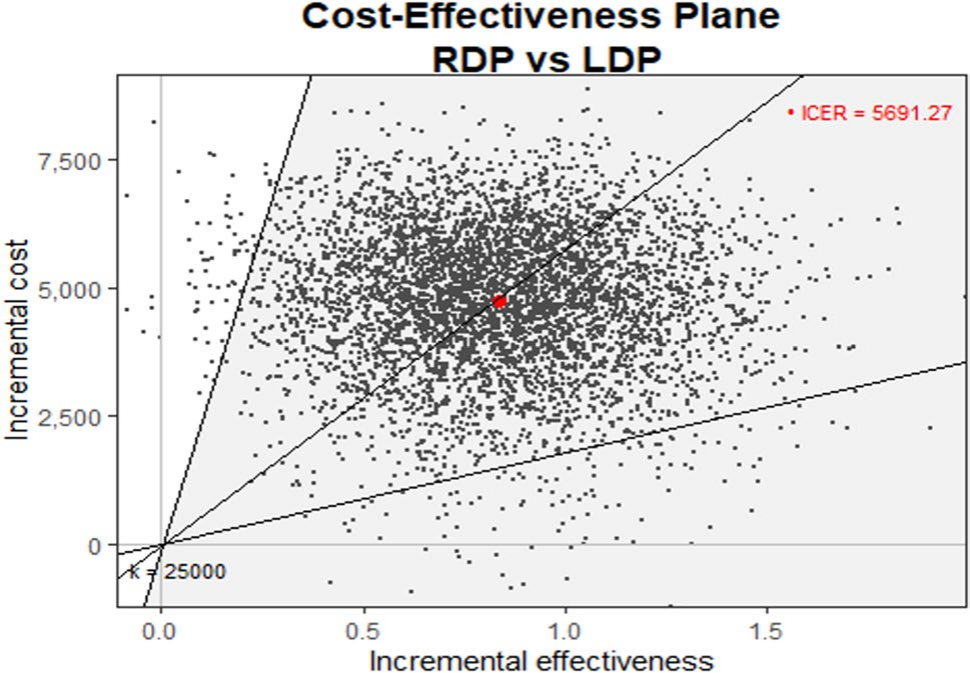
The cost-effectiveness plane. ICER slope was 5691 Euros (95% CI 1785–17,916 Euros)

**Fig. 3:**
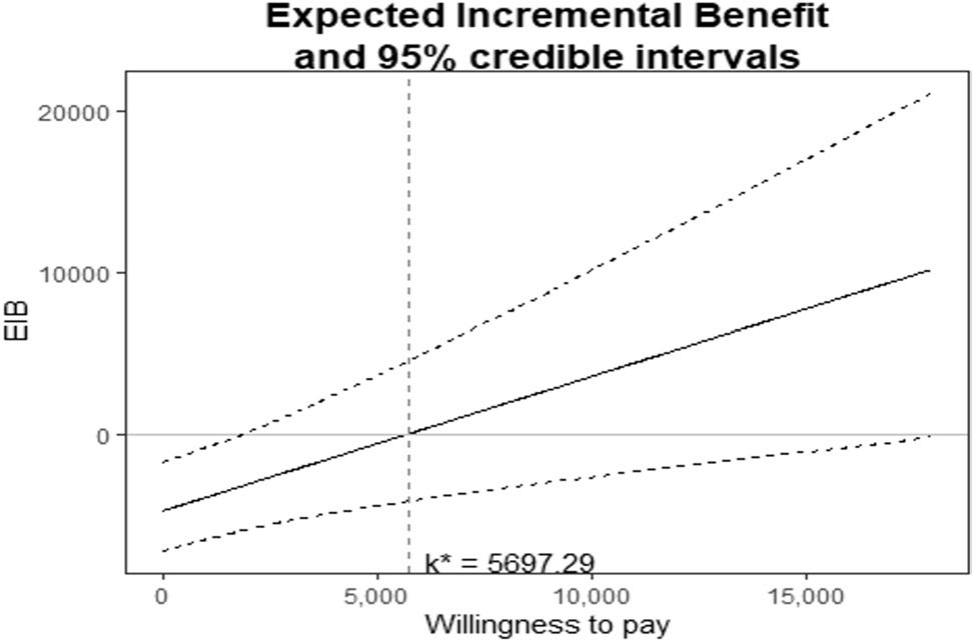
The expected incremental benefit (EIB) plot. The willingness to pay (WTP) was 5697 Euros

**Fig. 4:**
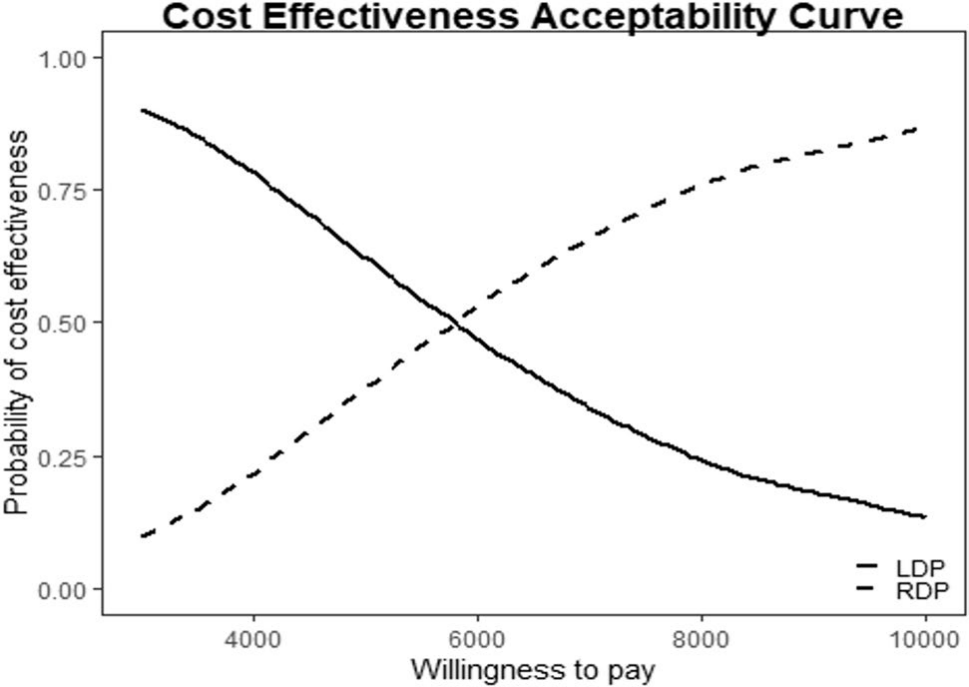
The cost-effectiveness acceptability curve (CEAC). The CEAC demonstrated that the robotic approach had a higher probability of being more cost-effective than the laparoscopic procedure when a WTP of at least 5697 Euros/QALY was accepted

All the information regarding the cost-effectiveness analysis was extracted from the EQ-5D. The RDP had a better mean QALY than the LDP group (*p* = 0.031). The cost-effectiveness plane is reported in Fig. 2. All observations were found to be in the uncertain quadrant (northeast). The incremental cost-effectiveness ratio (ICER) slope was 5691 Euros (95% CI 1785–17,916 Euros) for any additional QALY gain. The expected incremental benefit (EIB) plot is shown in Fig. 3. The willingness to pay (WTP) was 5697 Euros. Figure 4 reported the cost-effectiveness acceptability curve (CEAC). The CEAC demonstrated that the robotic approach had a higher probability of being more cost-effective than the laparoscopic procedure when a WTP of at least 5697 Euros/QALY was accepted.

### Quality of life analysis

**Fig. 5:**
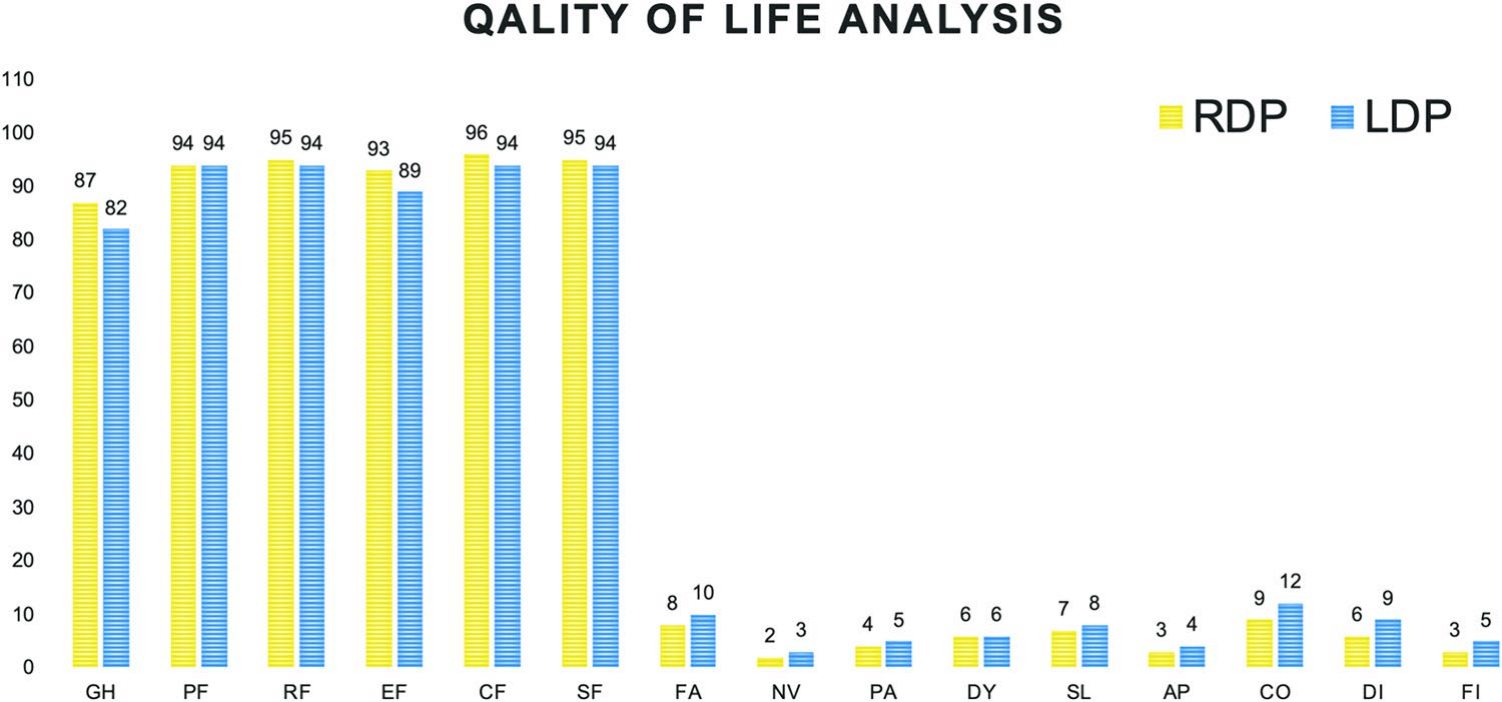
The Quality of Life analysis demonstrated a significant improvement in the RDP group in the global health (GH) and emotional functioning (EF) domains (*p* = 0.037 and *p* = 0.026, respectively)

Due to patient non-cooperation or loss to follow-up, the QoL analysis was performed on 446 patients (216 belonging to the LDP group and 230 to the RDP group). The median cohort follow-up was 68 months. The QoL analysis demonstrated a significant improvement in the RDP group over the postoperative period, which appears to be superior in all considered items, with significant advantages in the global health (GH) and emotional functioning (EF) domains (*p* = 0.037 and *p* = 0.026, respectively, Fig. 5).

### Intra- and postoperative data

When comparing the two techniques for intraoperative variables (Table 1), statistically significant differences were found in favor of RDP for conversion (14 vs. 4%, *p* < 0.001), splenic preservation rate (21 vs. 32%, *p* = 0.002). Higher laparoscopic multiorgan resections were reported (16 vs. 10%, *p* = 0.016). The pancreatic stump management varied between groups, with the stapler device being more frequently adopted in LDP (75 vs. 64%, *p* = 0.002). Lastly, the median operation time (OT min, median) was significantly higher in the RDP group compared with the LDP one (285 vs. 250 min, *p* < 0.001).

Postoperative outcomes are outlined in Table 3. More postoperative complications (66 vs. 56%, *p* = 0.009), with statistically significant higher rates of PPH (10 vs. 6%, *p* = 0.044) and intra-abdominal collections (and 25 vs. 18%, *p* = 0.027) were recorded in the LDP group. Whereas, no statistically significant differences were found for the other postoperative complications after the propensity score matching (*p* > 0.05). Pathologic data are shown in Table 4. After the propensity score matching the study population was balanced. Particularly, final pathology, tumor size, and lymph nodes harvested were comparable (*p* > 0.05). However, the oncological performance of the robotic technique, expressed by the R status, seemed to be superior to the laparoscopic approach (88 vs. 68%, *p* = 0.003).

**Table 3:** Postoperative outcomes

	Before propensity score-matching			After propensity score-matching		
	Laparoscopic DP	Robotic DP	*p*-value	Laparoscopic DP	Robotic DP	*p*-value
	(*n* = 271, 48%)	(*n* = 293, 52%)		(*n* = 159, 50%)	(*n* = 159, 50%)	
Postoperative complications, *n*° (%)	179 (66%)	164 (56%)	**0.009**	105 (66%)	99 (62%)	0.559
Clavien-Dindo Score ≥ grade III, *n*° (%)	33 (12%)	38 (13%)	0.438	23 (15%)	25 (16%)	0.876
POPF, *n*° (%)			0.536			0.624
Grade B	60 (22%)	64 (22%)		33 (21%)	39 (24%)	
Grade C	1 (0.4%)	3 (1%)		1 (0.6%)	3 (2%)	
PPH, *n*° (%)	26 (10%)	16 (6%)	**0.044**	20 (13%)	9 (6%)	0.051
DGE, *n*° (%)	2 (0.7%)	7 (2%)	0.109	1 (0.6%)	3 (2%)	0.623
Intrabdominal abscess, *n*° (%)	67 (25%)	52 (18%)	**0.027**	41 (26%)	29 (18%)	0.137
Chyle leak, *n*° (%)	4 (2%)	10 (3%)	0.113	3 (2%)	6 (4%)	0.502
Sepsis, *n*° (%)	14 (5%)	16 (5%)	0.514	8 (5%)	12 (8%)	0.488
Reintervention, *n*° (%)	16 (6%)	15 (5%)	0.411	16 (6%)	15 (5%)	0.411
Readmission, *n*° (%)	22 (8%)	32 (11%)	0.162	12 (8%)	17 (11%)	0.436
Length of hospital stay, days, median (IQR)	9 [7–13]	8 [6–12]	0.166	9 [6–14]	8 [7–12]	0.898
Mortality, *n*° (%)	2 (0.7%)	1 (0.3%)	0.471	2 (1%)	0 (0%)	0.498

Bold value indicate statistical significance (*p* < 0.05)

*POPF* postoperative pancreatic fistula, *DGE* delayed gastric empty, *PPH* post-pancreatectomy hemorrhage

**Table 4:** Pathologic data

	Before Propensity Score-Matching			After Propensity Score-Matching		
	Laparoscopic DP	Robotic DP	*p*-value	Laparoscopic DP	Robotic DP	*p*-value
	(*n* = 271, 48%)	(*n* = 293, 52%)		(*n* = 159, 50%)	(*n* = 159, 50%)	
Pathology, no. (%)			**0.016**			0.118
PDAC	73 (27%)	54 (18%)		40 (25%)	24 (15%)	
pNET	94 (35%)	92 (31%)		58 (37%)	53 (33%)	
IPMN	21 (8%)	18 (6%)		12 (8%)	12 (8%)	
SCN/MCN	52 (19%)	77 (26%)		29 (18%)	43 (27%)	
SPT	17 (6%)	21 (7%)		13 (8%)	14 (8%)	
Other	14 (5%)	31 (11%)		7 (4%)	13 (8%)	
Tumor size, mm, median (IQR)	28 [20–44]	30 [20–40]	0.583	28 [20–40]	30 [20–40]	0.608
Harvest lymph nodes, median (IQR)	19 [10–32]	15 [8–25]	**0.002**	18 [10–29]	14 [8–23]	0.054
R0 status*	47 (64%)	40 (74%)	0.166	28 (68%)	21 (88%)	**0.003**
Incisional hernia	4 (2%)	10 (3%)	0.205	2 (2%)	4 (4%)	0.690

Bold value indicate statistical significance (*p* < 0.05)

*Only PDAC patients

*PDAC* pancreatic ductal adenocarcinoma, *pNET* pancreatic neuroendocrine tumor, *IPMN* intraductal papillary mucinous neoplasm, *MCN* mucinous cystic neoplasm, *SCN* serous cystic neoplasm, *SPT* solid pseudopapillary tumor

## Discussion

This study investigated the cost-effectiveness, quality of life, and surgical outcomes of minimally invasive distal pancreatectomy (MIDP), comparing LDP versus RDP. Despite having higher intraoperative costs, RDP might be considered a cost-effective alternative if decision-makers and funders of healthcare accepted a willingness to pay more than 5697 Euros per quality-adjusted life-year (QALY).

Robotic-assisted pancreatectomy has been popularized over the last decade and has gained steady uptake thanks to its technical benefits, including enhanced dexterity, reduction of natural tremors, and increased surgical precision and vision. Different studies have demonstrated its feasibility, safety, and reproducibility [20, 21], with comparable perioperative results compared to LDP [22]. However, the cost of surgical instruments was still significantly more expensive for both the MI approach compared to open surgery [23, 24]. A recent meta-analysis pointed the light on this contributor to the raised costs. Indeed, has been demonstrated as the requirement for additional equipment such as laparoscopic and robotic electrosurgical instruments and needle drivers play a central role in the higher crude costs of the MI procedures, with robotic surgery being the most expensive, followed by laparoscopic and then open surgery [25, 26].

Despite the higher costs of the robotic procedure, the overall postoperative expenses were balanced without a significant difference (*p* = 0.200). The advantages of using a robotic platform during DP were evident in the intraoperative outcomes. The study results described a lower conversion rate in the RDP group (*p* < 0.001) and a comparable blood loss and need for blood transfusion (respectively *p* = 0.289 and *p* = 0.108) when compared with the LDP group. These benefits were also recorded in the analysis of the postoperative outcomes. The RDP group reported a lower overall occurrence of complications, PPH, and intra-abdominal abscess (*p* = 0.009, *p* = 0.044, and *p* = 0.027, respectively), reinforcing the concept that RDP seems to be superior to LDP in terms of short-term outcomes, even when the group had been balanced, possibly reducing the postoperative infectious complications that had widely recognized as major burden of morbidity after DP [27].

The oncological safety of MIDP has been widely investigated in the last few years. The DIPLOMA trial was designed to investigate the non-inferiority of MIDP compared to open distal pancreatectomy (ODP) regarding the microscopically radical resection rate of pancreatic ductal adenocarcinoma (PDAC) in an international setting [28]. Future steps should include a comparison of the oncological outcomes between RDP and LDP. However, the present results demonstrated that the rate of R0 resections when only PDAC patients are considered seems to be superior in the RDP compared to LDP (*p* = 0.003). Because the RDP group had more benign lesions and performed more spleen-preserving resections, the initially observed difference in harvested lymph nodes (significantly higher in LDP group, *p* = 0.002) was smoothed out when balancing the population.

Improved quality of life (QoL) and cosmetic satisfaction are often mentioned as a benefit of minimally invasive surgery [29]. Despite a few studies examining the difference between RDP and LDP in terms of quality of life, De Pastena et al. demonstrated that RDP was associated with improvements in specific domains compared to a matched LDP group of patients [30]. The present study is the first to analyze these aspects with a comparison between RDP and LDP in a multicentric setting, confirming that the RDP group generally performed better in all the considered items, specifically in the global health and emotional functioning domains. Because RDP showed the same surgical outcomes as LDP, it might be speculated that the differences in QoL items resulted from a somewhat favorable psychological impact of the robotic procedure. Indeed, the patient’s perception of receiving the newest and most innovative surgical treatment might enhance the recovery process after surgery (the length of stay was shorter but not statistically different, *p* = 0.898) and beyond the discharge period. This point was strictly correlated to the financial impact of the procedure, as patients who underwent RDP had a faster return to normal daily activities and work, with a reduction of the financial impact of surgery. The translation of that into economic benefit resulted in an increase of the mean QALY gained (higher in the RDP group). This was further strengthened by the potential QALY that could be obtained considering the life expectancy of the patients operated on with MIDP (typically young adults suffering from diseases with a favorable prognosis).

The strength of the present study lies in its novelty as it is the first cost-effectiveness analysis comparing RDP and LDP in a multicentric setting that involved four high-volume centers. As expected from the analysis of the crude costs, the robotic procedure was more expensive than the laparoscopic approach, consistent with previous reports (26). As published by Souche et al., the higher costs were related to the specific instrumentation used for RDP, which was inevitably more expansive than laparoscopic instrumentation. However, the present study demonstrated improvements in some QoL items and additional QALY gained compared to LDP. The CEAC (Fig. 3) showed that if society were willing to pay an additional 5697 Euros to the mean costs of the LDP, the RDP would have had more probability of gaining QALY. Furthermore, even if burdened by higher costs, the robotic procedure could be financially sustainable within the Eurozone compared to the Italian GDP per capita. Although previous studies reported an increase in the expenses without compensation in the short-term surgical outcomes [31], the results of the present study suggest the possible long-term benefits of RDP. However, the promising results and the economic sustainability of the robotic procedure in a Public Health System should be further investigated through a valid Health Technology Assessment on pancreatic surgery. Moreover, the use of the robotic device should be reserved for high-volume centers, where the standardization of the surgical technique and the surgical training could reduce the costs of the procedure.

The study has some major limitations. First, different regional reimbursements (two Italian regions were involved) could lead to a bias. Second, the present study did not evaluate the acquisition cost of laparoscopic instruments and robotic consoles. Third, the QLQ-C30 questionnaire is validated for use in patients with malignancies. Fourth, the selection process for patients seems to have been influenced more by the availability of the robot than by clinical, technical, or pathological factors. Fifth, the short follow-up does not allow us to speculate on any middle- to long-term results.

These limitations are unlikely to significantly affect the current findings. The technology costs could be overcome with different methods of acquisition, such as loan for use. However, the patient selection process limits the generalizability of the results to real-world scenarios, where robot availability might be less of a factor than clinical need.

## Conclusion

The present study demonstrates that RDP can be considered not inferior to LDP. The differences in surgical outcomes, global health status, and financial implications were not persuasive enough to declare the superiority of one surgical technique over the other. The widespread adoption of robotic technology in the future, within a competitive market and with potential ease of access and cost reduction, could enhance the benefits of this procedure by lowering the WTP. Until a Health Technology Assessment on pancreatic surgery is conducted, the use of RDP should be judiciously employed and reserved for high-volume centers within dedicated research programs.

## Data Availability

From the corresponding author upon reasonable request.
